# Respiratory physiology after resupination following prone ventilation to predict 28-day mortality in mechanically ventilated patients: a machine learning analysis

**DOI:** 10.1038/s41598-026-39336-3

**Published:** 2026-02-10

**Authors:** Lada Lijović, Tariq A. Dam, Moon Seong Baek, Tae Wan Kim, Gyungah Kim, Paul W. G. Elbers, Won-Young Kim, The Dutch ICU Data Sharing Collaborators, Diederik Gommers, Olaf L. Cremer, Rob J. Bosman, Sander Rigter, Evert-Jan Wils, Tim Frenzel, Dave A. Dongelmans, Remko de Jong, Marco A.A. Peters, Marlijn J.A Kamps, Dharmanand Ramnarain, Ralph Nowitzky, Arjan Cloïn, Wouter de Ruijter, Louise C. Urlings-Strop, Ellen G.M. Smit, D.Jannet Mehagnoul-Schipper, Tom Dormans, Cornelis P.C. de Jager, Stefaan H.A. Hendriks, Sefanja Achterberg, Evelien Oostdijk, Auke C. Reidinga, Barbara Festen-Spanjer, Gert B. Brunnekreef, Alexander D. Cornet, Walter van denTempel, Age D. Boelens, Peter Koetsier, Judith Lens, Harald J. Faber, A. Karakus, Robert Entjes, Paul de Jong, Thijs C.D. Rettig, Sesmu Arbous, Lucas M. Fleuren, Patrick J. Thoral

**Affiliations:** 1 Department of Intensive Care Medicine, Center for Critical Care Computational Intelligence, Amsterdam Medical Data Science, Amsterdam PublicHealth, Amsterdam CardiovascularScience, Amsterdam Institute for Infection and Immunity, Amsterdam UMC, University of Amsterdam, Vrije Universiteit, Amsterdam, The Netherlands; 2 Department of Anesthesiology, Intensive Care and Pain Management, University Hospital Center Sestre Milosrdnice, Zagreb, Croatia; 3 Division of Pulmonary and Critical Care Medicine, Department of Internal Medicine, Chung-Ang University Hospital, Chung-Ang University College of Medicine, Seoul, Republic of Korea; 4 Department of Intensive Care, Erasmus Medical Center, Rotterdam, The Netherlands; 5 Intensive Care, UMC Utrecht, Utrecht, The Netherlands; 6 OLVG, Amsterdam, The Netherlands; 7 Department of Anesthesiology and Intensive Care, St. Antonius Hospital, Nieuwegein, The Netherlands; 8 Department of Intensive Care, Franciscus Gasthuis & Vlietland, Rotterdam, The Netherlands; 9 Department of Intensive Care Medicine, Radboud University Medical Center, Nijmegen, The Netherlands; 10 Intensive Care, Bovenij Ziekenhuis, Amsterdam, The Netherlands; 11 Intensive Care, Canisius Wilhelmina Ziekenhuis, Nijmegen, The Netherlands; 12 Intensive Care, Catharina Ziekenhuis Eindhoven, Eindhoven, The Netherlands; 13 Department of Intensive Care, ETZ Tilburg, Tilburg, The Netherlands; 14 Intensive Care, HagaZiekenhuis, Den Haag, The Netherlands; 15 Intensive Care, Laurentius Ziekenhuis, Roermond, The Netherlands; 16 Department of Intensive Care Medicine, Northwest Clinics, Alkmaar, The Netherlands; 17 Intensive Care, Reinier de Graaf Gasthuis, Delft, The Netherlands; 18 Intensive Care, Spaarne Gasthuis, Haarlem en Hoofddorp, The Netherlands; 19 Intensive Care, VieCuri Medisch Centrum, Venlo, The Netherlands; 20 Intensive care, Zuyderland MC, Heerlen, The Netherlands; 21 Department of Intensive Care, Jeroen Bosch Ziekenhuis, Den Bosch, The Netherlands; 22 Intensive Care, Albert Schweitzerziekenhuis, Dordrecht, The Netherlands; 23 Haaglanden Medisch Centrum, Den Haag, The Netherlands; 24 Maasstad Ziekenhuis Rotterdam, Rotterdam, The Netherlands; 25 SEH, BWC, Martiniziekenhuis Groningen, The Netherlands; 26 Intensive Care, Ziekenhuis Gelderse Vallei, Ede, The Netherlands; 27 Department of Intensive Care, Ziekenhuisgroep Twente, Almelo, The Netherlands; 28 Department of Intensive Care, Medisch Spectrum Twente, Enschede, The Netherlands; 29 Department of Intensive Care, Ikazia Ziekenhuis Rotterdam, Rotterdam, The Netherlands; 30 Antonius Ziekenhuis Sneek, Sneek, The Netherlands; 31 Intensive Care, Medisch Centrum Leeuwarden, Leeuwarden, The Netherlands; 32 IJsselland Ziekenhuis, Capelle aan den IJssel, The Netherlands; 33 WZA, Assen, The Netherlands; 34 Department of Intensive Care, Diakonessenhuis Hospital, Utrecht, The Netherlands; 35 Department of Intensive Care, Adrz, Goes, The Netherlands; 36 Department of Anesthesia and Intensive Care, Slingeland Ziekenhuis, Doetinchem, The Netherlands; 37 Department of Anesthesiology and Intensive Care, Amphia Ziekenhuis, Breda, The Netherlands; 38 LUMC, Leiden, The Netherlands

**Keywords:** Biomarkers, Diseases, Health care, Medical research, Physiology, Risk factors

## Abstract

The clinical significance of resupination parameters following prone positioning remains largely unknown. This study employed machine learning to predict the survival of patients receiving mechanical ventilation (MV) by analyzing oxygenation and respiratory mechanics after resupination. Data were extracted from the COVID-Predict Dutch Data Warehouse. Patients receiving MV who underwent the supine–prone–supine sequence were selected, and the variables related to respiratory physiology within 4 h before proning and after resupination were recorded. Machine learning models were trained on the features selected using LASSO regression to predict the 28-day mortality. Patients who did not survive (157/522, 30.1%) had lower PaO_2_/FiO_2_ values, higher ventilatory ratios, increased physiological dead space, higher driving pressure, and lower static and dynamic lung compliance values at resupination. The predictive performance of the individual clinical parameters for 28-day mortality was generally modest, and FiO_2_, PaO_2_/FiO_2_, physiological dead space, and dynamic lung compliance were the best predictors of mortality. Overall, XGBoost showed the best recall (0.732) and maintained the highest AUC (0.719), while its F1-score (0.500) was the best for predicting mortality despite a low precision (0.380). Survival after prone positioning in patients receiving MV can be stratified by physiological responses after resupination.

**Supplementary Information:**

The online version contains supplementary material available at 10.1038/s41598-026-39336-3.

## Introduction

Acute respiratory distress syndrome (ARDS) affects approximately 10% of patients admitted to the intensive care unit (ICU) and 25% of those requiring mechanical ventilation (MV), with a mortality rate ranging from 30% to 50%^1^. Prone positioning can improve outcomes in patients with moderate-to-severe ARDS by improving gas exchange, altering lung mechanics to facilitate lung-protective ventilation, and possibly mitigating deleterious effects of ARDS and MV on the circulation^2–4^.

Meanwhile, prone positioning is time-consuming, labor-intensive, and requires a team of skilled medical professionals^5^. Additionally, complications such as pressure sores or endotracheal tube obstruction are more common in the prone position than in the supine position^6^. Prolonged prone positioning can also cause atelectasis in the dependent region of the lungs^7^. Thus, accurately determining the response to prone positioning may be valuable for a timely decision on continuing or terminating the session. The prone position improves oxygenation in most, but not all, patients with ARDS^2,3^. Several studies have assessed whether the oxygenation response during prone positioning predicts mortality in ARDS; however, the findings are inconsistent^8–10^. Nonetheless, clinicians often rely solely on changes in oxygenation during prone positioning, overlooking potential benefits that may protect the lungs and heart even without significant changes in oxygnation.

We hypothesized that the response to resupination might contain more valuable information on the decision to stop or continue proning sessions. Although PaO_2_/FiO_2_ is the most commonly used and evidence-based criteria, other respiratory physiology factors such as ventilatory ratio, driving pressure, respiratory system compliance, and mechanical power should also be monitored to optimize gas exchange while minimizing ventilator-induced lung injury^11^. The rationale is that sustained improvement in these parameters following resupination might be associated with improved clinical outcomes, thereby considering subsequent prone session. Otherwise, other rescue therapies, such as inhaled nitric oxide or extracorporeal membrane oxygenation, should be implemented. However, this has yet been subject to investigation.

To contribute to bridging this knowledge gap, we used machine learning to assess the value of respiratory physiology following resupination to predict survival in patients receiving MV who underwent proning. Recently, machine learning has been used in ARDS to classify severity, identify phenotypes, and assess subgroups exhibiting differential responses to treatment^12–14^. We hypothesized that machine learning techniques known for their superior classification and prediction performance could be applied to highly granular electronic health record (EHR) data to distinguish between survivors and non-survivors.

## Methods

### Covid-predict dutch data warehouse

Data for the study were extracted from the COVID-Predict Dutch Data Warehouse, a multicenter EHR database with complete admission data of critically ill patients with coronavirus disease 2019 (COVID-19) from 25 hospitals in the Netherlands^15^. An extract-transform-load (ETL) pipeline was developed to collect and process data from local EHR systems across multiple hospitals. Customized structured query language queries extracted data including patient demographics, clinical observations, medications, and vital signs, ensuring consistency and privacy through Secure Hash Algorithm (SHA-256) pseudonymization and secured CSV transfer. During the transformation phase, data were harmonized by mapping raw parameters to a common vocabulary of 942 clinically relevant terms, supplemented by Logical Observation Identifiers Names and Codes and Systematized Nomenclature of Medicine Clinical Terms standards^16,17^. Manual mapping resolved nomenclature discrepancies and standardized units, consolidating similar measurements (e.g., various temperature readings into a single “temperature” variable). Data on additional clinical parameters such as ventilatory ratio were derived, and complex events such as intubation were identified using predefined algorithms^18^.

The transformed data were loaded into a structured database organized into domain-specific tables (e.g., patient demographics and clinical observations). Data enrichment included calculating derived clinical scores such as the Sequential Organ Failure Assessment score^19^. Throughout the ETL process, data quality was continuously validated by checking completeness, verifying parameter mapping, and comparing clinical scores to the national benchmarks. Anomalies were identified through distribution plots, and inconsistencies were resolved by cross-referencing with the data of the original hospital. The Medical Ethics Committee at Amsterdam UMC waived the need for informed patient consent and approved the opt-out procedure for the collection of data of patients with COVID-19 during the COVID-19 crisis, as documented under number 2020.156. Study procedures were followed in accordance with the ethical standards of the responsible committee on human experimentation and with the Helsinki Declaration of 1975.

### Cohort extraction

Data were extracted from the processed admission records of patients on intubation in the ICU aged > 18 years with complete information on age and sex (March 2020–February 2021). Only the first admission episodes were included in this study. Patients were included if they experienced the specific sequence of body positioning from supine to prone and back to supine (supine–prone–supine) identified through timestamped records, ensuring that this sequence overlapped with the first MV period. The left and right decubital positions were considered supine. Subsequent prone sessions were not analyzed, and patients with no record of resupination were excluded. Patients were also excluded if the position sequence did not coincide with their initial MV period or if the duration of prone positioning exceeded 24 h. In this study, ARDS could not be diagnosed according to the Berlin criteria^20^ or the global definition^21^ due to lack of imaging data.

### Data preprocessing

Laboratory and ventilatory parameters were extracted for each patient and subsequently processed to address the outliers. Outliers were identified through distribution analysis, and predefined manual thresholds were applied for unrealistic clinical values. For variables representing the supination phase, data were selected based on the closest available values to the timestamp marking the initiation of prone positioning, with a restriction that no values recorded > 4 h before the timestamp were included. For multiple values within a specified timeframe (e.g., SpO_2_, tidal volume), the mean was calculated to represent the variable due to the high granularity that followed a normal distribution. Whereas in other cases (pH, aspartate aminotransferase, alanine aminotransferase, PaO_2_), only a single value existed. Similarly, for variables associated with the resupination phase, data were selected based on the closest available values to the timestamp of resupination commencement, excluding values recorded > 4 h after the timestamp. Given that clinical practice introduces some variance in the timing of measurements, these timeframes were chosen to accommodate potential delays while ensuring data relevance to the corresponding clinical events.

### Variables and outcomes

Demographic data and data on comorbidities were extracted at baseline. Laboratory values were extracted at admission, including the first value recorded within 12 h of admission. The ventilatory ratio was calculated as follows: (minute ventilation × PaCO_2_)/(predicted body weight × 100 × 37.5)^22^. Physiological dead space was calculated using the Bohr equation as follows: dead space volume/tidal volume = (PaCO_2_ – expired CO_2_ partial pressure)/PaCO_2_^23^. The recorded dynamic compliance was calculated as tidal volume/(peak pressure – PEEP [positive end-expiratory pressure]), and the recorded static compliance was calculated as tidal volume/driving pressure. The mechanical power was calculated as tidal volume × (peak pressure – [0.5 × driving pressure]) × respiratory rate × 0.1^24^. When only PEEP and pressure above PEEP were available, it was calculated as tidal volume × (PEEP + pressure above PEEP) × respiratory rate × 0.098, where the pressure above PEEP was defined as the peak pressure – PEEP^25^. All variables were evaluated for their availability and completeness in the dataset, ensuring that the selection was based on both clinical significance and data quality.

The primary outcome was death at 28 days, defined as mortality within 28 days of the start of MV. Mortality was chosen as the primary outcome measure because it is objective, patient-centered, and a commonly used endpoint in previous studies evaluating the response to prone positioning among patients with ARDS^8–10,26,27^.

### Statistical analysis

The normality of continuous variables was assessed using the Shapiro–Wilk test. Continuous variables were summarized as mean ± standard deviation or median (Q1, Q3) and compared using the Student’s t-test or Mann–Whitney *U* test, as appropriate. Qualitative variables were summarized using frequencies and percentages and compared using the chi-square (χ^2^) or Fisher’s exact tests (for frequencies < 5). A missingness threshold of 50% was considered inadequate, and the data were not imputed in the baseline evaluation. To establish a baseline for model comparison and assess the predictive potential of the initial feature set, a preliminary evaluation was performed using a single 70-30 train–test split. The predictor variables selected for the machine learning model were imputed using multivariate imputation by chained equations^28^. The area under the receiver operating characteristic curve (AUC-ROC) was used as a performance metric for the 28-day mortality. To specifically address multicollinearity and limit the predictor set, feature selection was performed using LASSO regression, an L1-penalized model that shrinks coefficients and can set some to zero, thereby performing variable selection^29^. LASSO model was trained on a wide range of potential alpha values generated logarithmically between 10^–6^ and 10, with 5-fold cross-validation and 10,000 iterations (see Supplementary Fig. S1)^30^. For the machine learning models, logistic regression, a grid-search optimized XGBoost classifier, and a decision tree were trained on the features selected by LASSO, incorporating class weights to address potential class imbalances^31,32^. The final model performance was evaluated on the held-out test set using the AUC-ROC, precision recall, and F1-scores for 28-day mortality. For extraction of coefficients for variables in the final logistic regression model, Statsmodels package was used^33^.

Data analysis and model development were performed using Python (v3.10.12). Data manipulation and preprocessing were conducted using Pandas (v2.2.1) and NumPy (v1.26.4). For the machine learning pipeline, including feature scaling and logistic regression, we utilized Scikit-learn (v1.4.1), while the XGBoost classifier was optimized using XGBoost (v2.0.3). Formal statistical inference for the regression models was conducted using Statsmodels (v0.14.1) and SciPy (v1.12.0). All visualizations were generated with Matplotlib (v3.8.3).

## Results

**Fig. 1:**
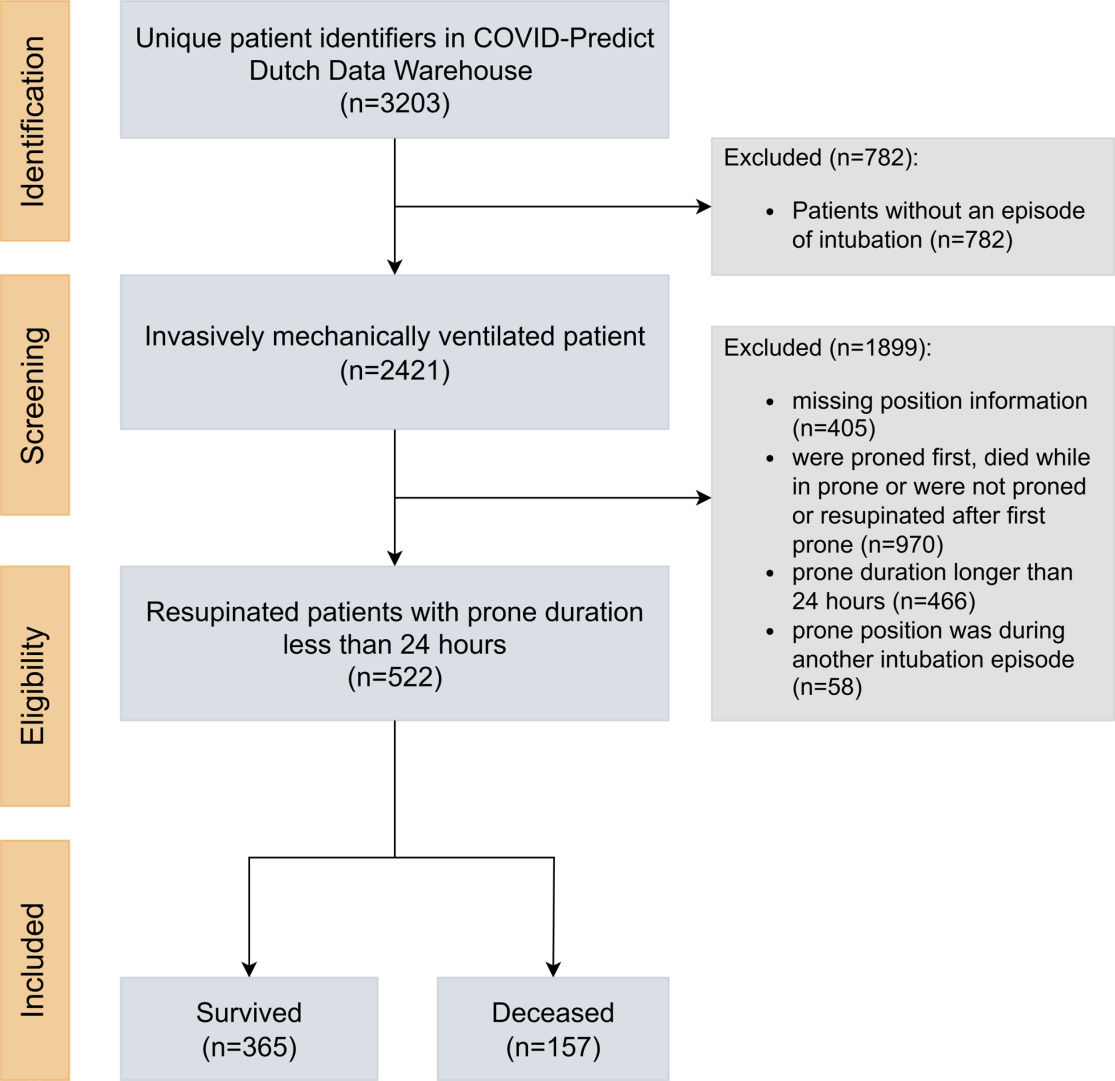
Cohort selection process from the COVID-Predict Dutch Data Warehouse. COVID: coronavirus disease.

During the study period, 2,421 of 3,203 (75.6%) patients in the original database received MV during their ICU admission and were reviewed for eligibility (Fig. 1). After excluding 405 patients with missing position data, 522 of 2,016 (25.9%) patients were resupinated after less than 24 h of initial prone positioning during the first MV period. Of these, 365 (69.9%) survived and 157 (30.1%) died within 28 days of MV initiation. A comparison of demographic data, vital parameters, and laboratory values at admission according to 28-day mortality is shown in Table 1.

**Table 1:** Demographic data, vital parameters, and laboratory values according to the 28-day mortality.

Variable	Data availability (%)	Survivors (n = 365)	Non-survivors (n = 157)	*P*-value
Age, years	100.0	63 (56, 70)	70 (64, 74)	< 0.001
Male sex	99.6	277 (75.9)	118 (75.2)	0.87
Body mass index, kg/m^2^	64.5	27.8 (25.3, 31.6)	27.4 (24.9, 30.3)	0.31
Comorbidities				
Diabetes	69.2	69 (26.2)	22 (22.5)	0.59
Cardiovascular insufficiency	66.1	5 (2.0)	3 (3.2)	0.80
COPD	66.3	24 (9.5)	9 (9.6)	> 0.99
Cirrhosis	66.1	0	0	
Chronic renal insufficiency	65.9	12 (4.8)	7 (7.5)	0.45
Neoplasm	66.1	0	2 (2.1)	0.13
Hematologic malignancy	66.3	4 (1.6)	3 (3.2)	0.62
Immunodeficiency	66.5	16 (6.4)	11 (11.6)	0.16
SOFA score	45.1	6 (5, 8)	7 (4, 9)	0.63
Vital parameters				
Body temperature, °C	92.1	37.4 (36.7, 38.3)	37.2 (36.3, 38.1)	0.03
Heart rate, /min	96.0	91 (78, 103)	88 (76, 109)	0.96
Mean arterial pressure, mmHg	95.6	87 (75, 99)	81 (72, 94)	0.005
SpO_2_, %	96.0	94.0 (91.0, 96.6)	93.0 (91.0, 95.0)	0.005
Laboratory values				
Leukocytes, × 10^9^/L	83.7	9.2 (6.7, 12.3)	10.4 (7.3, 13.9)	0.03
Hemoglobin, g/dL	91.0	7.7 (7.0, 8.5)	8.0 (6.9, 8.7)	0.60
Thrombocytes, × 10^9^/L	88.9	249 (182, 321)	231 (174, 304)	0.13
Creatinine, µmol/L	85.6	76.0 (62.0, 101.8)	90.0 (68.5, 122.0)	< 0.001
Total bilirubin, µmol/L	71.0	8.0 (6.0, 10.5)	8.0 (5.0, 12.0)	0.90
Sodium, mmol/L	92.2	137 (134, 140)	137 (135, 141)	0.30
Potassium, mmol/L	93.1	4.0 (3.6, 4.4)	4.0 (3.7, 4.6)	0.07
C-reactive protein, mg/L	74.3	172 (104, 267)	178 (126, 259)	0.21
Procalcitonin, ng/mL	24.8	0.4 (0.2, 0.7)	0.8 (0.3, 1.7)	0.001
Glucose, mmol/L	94.2	7.6 (6.5, 10.1)	8.4 (6.8, 12.0)	0.004
Arterial pH	82.5	7.41 (7.34, 7.46)	7.36 (7.30, 7.45)	0.001
Bicarbonate, mmol/L	55.5	24.6 (23.0, 27.3)	24.1 (21.2, 26.3)	0.03
Lactate, mmol/L	64.7	1.2 (1.0, 1.5)	1.4 (1.1, 1.8)	0.001

Data are presented as medians (Q1, Q3) or percentages. COPD: chronic obstructive pulmonary disease; SOFA: Sequential Organ Failure Assessment; SpO_2_: peripheral oxygen saturation.

The duration in the prone position was 17.0 (12.9, 20.2) h for survivors and 16.1 (10.3, 19.5) h for non-survivors (*P* = 0.12). Gas exchange and ventilatory parameters before prone positioning were generally similar between the groups (Table 2). In the resupinated position, non-survivors exhibited lower values of arterial pH (7.34 [7.3, 7.4] vs 7.37 [7.3, 7.4]; *P* = 0.006) and PaO_2_/FiO_2_ (118 [98, 141] vs 142 [118, 179]; *P* < 0.001) compared to survivors (Table 2). Non-survivors also had a median higher ventilatory ratio (0.21 [0.2, 0.3] vs 0.18 [0.2, 0.2]; *P* = 0.006) and increased physiological dead space (0.3 [0.2, 0.4] L vs 0.2 [0.1, 0.3] L; *P* < 0.001). For ventilatory parameters, non-survivors required a higher respiratory rate (24 [20, 28] /min vs 22 [20, 25] /min; P = 0.01), FiO_2_ (61 [52, 72]% vs 52 [43, 62]%; *P* < 0.001), and driving pressure (13.4 [10.0, 16.1] cmH_2_O vs 12.0 [9.3, 14.6] cmH_2_O; *P* = 0.04). The values of static and dynamic lung compliances were also lower in non-survivors (static: 32.6 [24.1, 46.7] cmH_2_O vs 38.2 [28.7, 50.1] cmH_2_O; *P* = 0.03, dynamic: 28.0 [21.4, 34.1] cmH_2_O vs 33.3 [25.8, 42.3] cmH_2_O; *P* < 0.001).

**Table 2:** Gas exchange and ventilatory parameters before prone and after resupine positioning according to the 28-day mortality.

Variable	Before prone				After resupine			
	Data availability (%)	Survivors (n = 365)	Non-survivors (n = 156)	*P*-value	Data availability (%)	Survivors (n = 365)	Non-survivors (n = 156)	*P*-value
**Gas exchange**								
Arterial pH	73.3	7.38 (7.3, 7.4)	7.36 (7.3, 7.4)	0.01	65.1	7.37 (7.3, 7.4)	7.34 (7.3, 7.4)	0.006
PaCO_2_, mmHg	77.4	46.0 (41.0, 54.4)	46.5 (40.5, 56.7)	0.39	67.6	49.5 (43.0, 57.0)	49.0 (42.0, 62.8)	0.58
PaO_2_, mmHg	79.7	70.0 (64.5, 77.3)	70.7 (62.7, 78.0)	0.77	67.7	72.8 (65.0, 81.0)	69.0 (62.6, 79.5)	0.02
PaO_2_/FiO_2_	78.1	125 (99, 153)	117 (94, 144)	0.09	69.5	142 (118, 180)	118 (99, 141)	< 0.001
Ventilatory ratio	54.9	0.17 (0.1, 0.2)	0.19 (0.2, 0.2)	0.02	47.4	0.18 (0.2, 0.2)	0.21 (0.2, 0.3)	0.006
Physiological dead space, L	68.7	0.2 (0.1, 0.3)	0.3 (0.1, 0.4)	0.001	60.6	0.2 (0.1, 0.3)	0.3 (0.2, 0.4)	< 0.001
**Ventilatory parameters**								
Respiratory rate, /min	84.8	24 (21, 27)	24 (22, 28)	0.07	73.7	22 (20, 25)	24 (20, 28)	0.01
FiO_2_, %	98.3	60 (50, 72)	65 (57, 74)	0.003	97.5	52 (43, 62)	61 (52, 72)	< 0.001
Tidal volume, mL	97.9	450 (401, 494)	443 (386, 496)	0.51	96.4	447 (400, 499)	450 (383, 503)	0.67
Minute ventilation, L/min	96.4	10.2 (8.9, 11.9)	10.7 (9.0, 12.4)	0.13	93.5	10.2 (8.9, 11.7)	10.6 (9.0, 12.6)	0.13
PEEP, cmH_2_O	97.5	13 (10, 15)	12 (10, 14)	0.08	96.4	12 (10, 15)	12 (10, 14)	0.33
Peak pressure, cmH_2_O	94.3	27.0 ± 5.3	27.5 ± 6.0	0.45	92.5	26.6 ± 5.7	27.8 ± 6.3	0.053
Driving pressure, cmH_2_O	80.8	12.4 (10.0, 15.1)	13.0 (10.0, 16.0)	0.25	78.9	12.0 (9.3, 14.6)	13.4 (10.0, 16.1)	0.04
Respiratory system compliance								
Dynamic, mL/cmH_2_O	77.4	31.3 (25.3, 40.5)	30.2 (22.4, 36.2)	0.02	73.5	33.3 (25.8, 42.3)	28.0 (21.4, 34.1)	< 0.001
Static, mL/cmH_2_O	79.6	34.5 (28.0, 49.0)	33.0 (25.0, 46.9)	0.36	76.8	38.2 (28.7, 50.1)	32.6 (24.1, 46.7)	0.03
Mechanical power, J/min	79.1	31.2 (24.1, 40.3)	31.0 (23.6, 38.7)	0.63	78.7	30.3 (22.8, 40.3)	32.3 (24.0, 42.7)	0.31

Data are presented as means ± standard deviation, median (Q1, Q3), or percentages. FiO_2_: fraction of inspired oxygen; PaCO_2_: partial pressure of arterial carbon dioxide; PaO_2_: partial pressure of arterial oxygen; PEEP: positive end-expiratory pressure.

Regarding resupine–preprone position-related differences, survivors demonstrated greater increases in PaO_2_/FiO_2_ (15 [–13, 50] vs –2 [–21, 22]; *P* < 0.001) and greater decreases in FiO_2_ (–7 [–17, 2]% vs –1 [–11, 7]%; *P* < 0.001; Table 3). Moreover, survivors showed a greater decrease in peak pressure (–0.5 [–2.8, 1.5] cmH_2_O vs 0.2 [–1.7, 2.4] cmH_2_O; *P* = 0.04). However, no significant differences were found between survivors and non-survivors with respect to changes in other gas exchange or ventilatory parameters. In survivors, the increase in PaO_2_/FiO_2_ to ≥ 20 was more frequent than that in non-survivors (28.8% vs 19.9%; *P* = 0.046), whereas an increase to < 20 was more common in non-survivors (48.1% vs 34.5%; *P* = 0.005).

**Table 3:** Differences in resupine–preprone position parameters in survivors and non-survivors according to 28-day mortality.

Variable	Data availability (%)	Survivors (n = 365)	Non-survivors (n = 156)	*P*-value
Δ Arterial pH	73.3	–0.01 (–0.05, 0.04)	–0.02 (–0.06, 0.04)	0.31
Δ PaCO_2_, mmHg	63.7	2.2 (–3.0, 8.0)	3.4 (–3.9, 9.2)	0.62
Δ PaO_2_, mmHg	65.5	0.4 (–0.9, 10.1)	0 (–10.5, 10.0)	0.47
Δ PaO_2_/FiO_2_	64.7	15 (–13, 50)	–2 (–21, 22)	< 0.001
Δ Ventilatory ratio	44.3	0.01 (–0.02, 0.03)	0.01 (–0.01, 0.03)	0.96
Δ Physiological dead space, L	58.2	0.02 (0.02, 0.1)	0.02 (–0.1, 0.1)	0.77
Δ Respiratory rate, /min	71.6	0 (–3, 2)	0 (–4, 2)	0.76
Δ FiO_2_, %	96.5	–7 (–17, 2)	–1 (–11, 7)	< 0.001
Δ Tidal volume, mL	95.4	–1 (–39, 39)	0 (–41, 47)	0.82
Δ Minute ventilation, L/min	92.1	0.03 (–1.0, 1.0)	0.1 (–0.9, 1.3)	0.42
Δ PEEP, cmH_2_O	95.4	0 (–1, 1)	0 (–1, 1)	0.44
Δ Peak pressure, cmH_2_O	91.0	–0.5 (–2.8, 1.5)	0.2 (–1.7, 2.4)	0.04
Δ Driving pressure, cmH_2_O	72.4	–0.2 (–2.0, 1.2)	0 (–1.8, 2.4)	0.26
Δ Dynamic compliance, mL/cmH_2_O	66.8	0.7 (–3.1, 4.0)	–0.2 (–4.4, 2.8)	0.12
Δ Static compliance, mL/cmH_2_O	70.6	0.5 (–6.1, 8.2)	0.5 (–6.2, 8.3)	0.90
Δ Mechanical power, J/min	77.0	–0.1 (–5.0, 5.1)	1.9 (–4.3, 8.4)	0.09
PaO_2_/FiO_2_ increase, mmHg				
≥ 20	64.7	105 (28.8)	31 (19.9)	0.046
< 20	64.7	126 (34.5)	75 (48.1)	0.005
PaCO_2_ decrease, mmHg				
≥ 1	63.7	127 (34.8)	60 (38.5)	0.48
< 1	63.7	99 (27.1)	46 (29.5)	0.66

Data are presented as medians (Q1, Q3) or percentages. FiO_2_: fraction of inspired oxygen; PaCO_2_: partial pressure of arterial carbon dioxide; PaO_2_: partial pressure of arterial oxygen; PEEP: positive end-expiratory pressure.

**Fig. 2:**
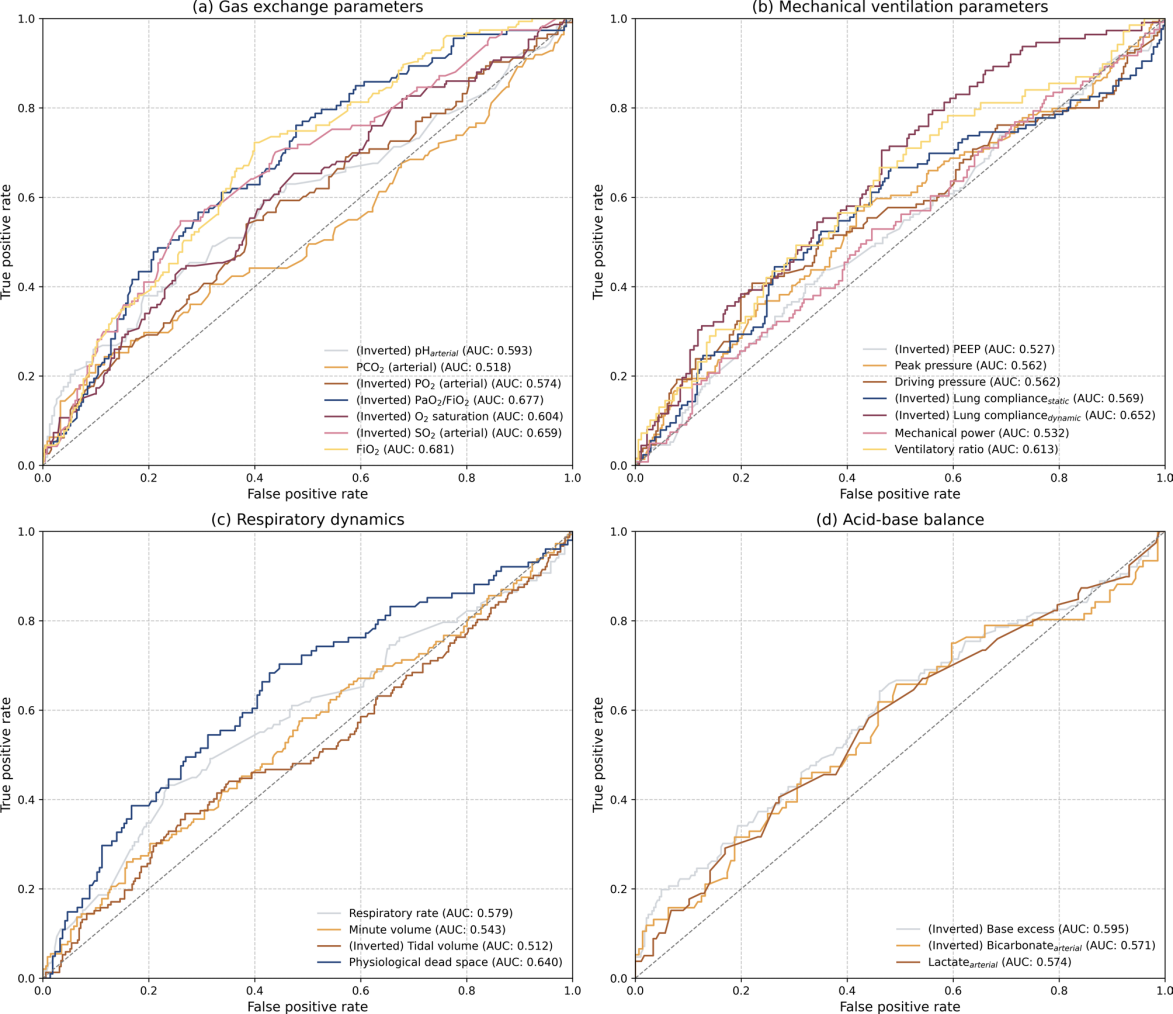
Receiver operating characteristic (ROC) curves for various physiological variables measured within 4 h after patients were resupinated following prone positioning to predict 28-day mortality. The curves are grouped by physiological type for (**a**) gas exchange parameters, (**b**) mechanical ventilation parameters, (**c**) respiratory dynamics, and (**d**) acid–base balance. For some variables, the initial ROC analysis showed AUCs < 0.5, indicating that higher values were associated with lower 28-day mortality (inverse relationship). To facilitate a visual comparison of the strength of the prediction signal for all variables on a similar scale, the prediction scores for these variables were inverted and labeled accordingly. AUC: area under the curve; FiO_2_: fraction of inspired oxygen; PaO_2_: partial pressure of arterial oxygen; PCO_2_: partial pressure of carbon dioxide; PEEP: positive end-expiratory pressure; SO_2_: oxygen saturation.

In the single held-out test, ROC analysis was used to assess the ability of 4-h post-resupination values to predict the 28-day mortality. For visualization purposes, the scores of variables with initial AUC < 0.5 were inverted (labeled accordingly; AUC reflects the strength of the inverse relationship; Fig. 2). Among the gas exchange parameters, FiO_2_ (AUC = 0.681) and PaO_2_/FiO_2_ (AUC = 0.677) showed the strongest inverse correlation, whereas PaCO_2_ showed the weakest correlation (AUC = 0.518). Physiological dead space also demonstrated a relatively high correlation (AUC = 0.640). For the MV parameters, dynamic lung compliance showed the strongest inverse correlation (AUC = 0.652). The AUCs for peak pressure and driving pressure were modest (both 0.562). Other respiratory dynamics and acid–base balance parameters demonstrated limited discriminatory power.

**Fig. 3:**
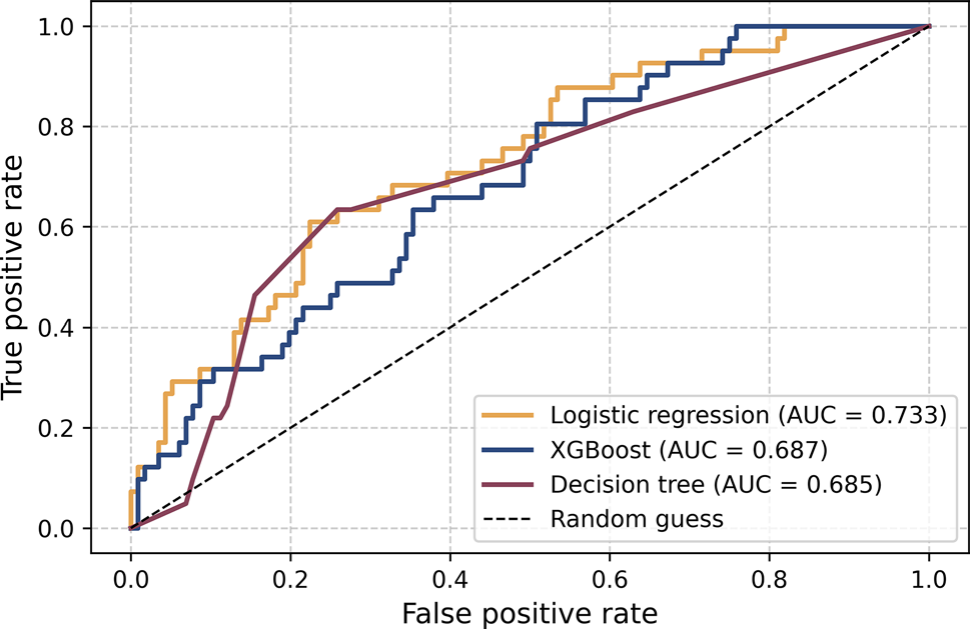
Receiver operating characteristic curves for 28-day mortality prediction models for the test dataset. AUC: area under the curve.

The 4-h post-resupination values were used to predict 28-day mortality after LASSO feature selection (see Supplementary Fig. S1). The coefficients for each variable selected in the logistic regression analysis are provided in Supplementary Table S1. After 0.7:0.3 split, there were 365 patients in the train dataset (249 survivors and 116 non-survivors) and 157 patients in the test dataset (116 survivors and 41 non-survivors). The AUC-ROC values indicated moderate discriminatory power (0.677–0.719), with XGBoost performing slightly better (0.719; see Supplementary Table S2 and Fig. 3). The accuracy was highest in logistic regression (0.771) and lowest in XGBoost (0.618). The precision values were generally low (0.380–0.619), whereas the recall value was the highest for XGBoost (0.732) and the lowest for logistic regression (0.317). The balanced F1-score was the highest for XGBoost (0.500). Overall, although all the models showed moderate discriminative power, XGBoost provided the best balance between precision and recall for mortality prediction after LASSO selection.

## Discussion

To the best of our knowledge, this is the first study to employ machine learning techniques to predict prone positioning-related survival in patients receiving MV using the post-resupination gas exchange and ventilatory parameters. After resupination, significant differences were observed between the 28-day survivors and non-survivors in terms of PaO_2_/FiO_2_, ventilatory ratio, physiological dead space, driving pressure, and lung compliance. The performance of machine learning models that used these values to distinguish between survivors and non-survivors was moderate.

In previous studies, sustained improvement in PaO_2_/FiO_2_ upon return to the supine position was independently associated with liberation from MV and survival^27,34^. However, as there was no improvement in other gas exchange or ventilatory parameters, it was difficult to establish a causal relationship between the oxygenation response after resupination and better outcomes. Conversely, the 4-h post-resupination values showed that patients who did not survive for 28 days had a higher ventilatory ratio, physiological dead space, respiratory rate, and driving pressures along with lower static and dynamic lung compliance values compared to survivors. Furthermore, the physiological dead space and dynamic lung compliance showed relatively strong correlations with mortality. Although a causal relationship cannot be established, the current results better reflect the underlying pathophysiology and clinical outcomes of resupination. Dead space might play an important role in predicting death in patients with ARDS^35^. The higher and sustained ventilatory ratios observed in non-survivors might indicate a severe ventilation/perfusion mismatch that cannot be reversed in the prone position^36^. Conversely, since chest wall compliance is expected to be reduced in the prone position, increased lung compliance at resupination would suggest a net gain in alveolar recruitment among survivors^3^. These findings suggest that resupination parameters may provide comprehensive information for interpreting proning response and patient prognosis. The decrease in PaCO_2_ due to better oxygenation also indicates that prone positioning induced lung recruitment. However, no significant differences were observed in resupination PaCO_2_ between survivors and non-survivors (49.5 [43.0, 57.0] vs 49.0 [42.0, 62.8] mmHg; *P* = 0.58), and it showed the lowest correlation with mortality. Although mechanical power in the prone position is reduced in patients with ARDS^37^, no significant differences were observed in mechanical power between survivors and non-survivors after resupination (30.3 [22.8, 40.3] J/min vs 32.3 [24.0, 42.7] J/min; *P* = 0.31). Additional studies are needed to better understand how to incorporate PaCO_2_ and mechanical power at resupination into the risk analysis for proning outcomes.

Although the oxygenation response immediately following prone positioning is commonly used to determine whether additional sessions should be continued, the association between a positive response and better outcomes has not been universally demonstrated. In the PROSEVA trial, which revealed a significant survival advantage of prone positioning, the survival benefit was not limited to patients who showed a physiological response to prone positioning^2,9^. Conversely, a retrospective cohort study emphasized that improved oxygenation after prone positioning (53% increase in PaO_2_/FiO_2_) improved the survival of patients with moderate to severe ARDS^10^. Several studies on patients with COVID-19 receiving MV have shown that proning outcomes are better in patients who respond to oxygenation than those in non-responders^8,34,38^. However, a recent machine learning study predicting responders to prone positioning in patients with COVID-19 found a weak association between PaO_2_/FiO_2_ response and survival^5^. Importantly, few of these studies have evaluated ventilatory parameters. Using PaO_2_/FiO_2_ alone as a single criterion during the prone position may be an oversimplified approach to adequately reflect the response to the maneuver.

The most appropriate time to assess the proning response might be after returning to the supine position. Numerous studies have revealed that the improved oxygenation from prone positioning does not persist after resupination^39,40^ and could be associated with worse outcomes. Indeed, the present results support this hypothesis; in patients who did not survive for 28 days, PaO_2_/FiO_2_ was lower after resupination, with the highest inverse correlation with mortality. While using PaO_2_/FiO_2_ to decide whether to continue or discontinue prone positioning, the FiO_2_ from which the ratio is calculated must be considered. For instance, if PaO_2_/FiO_2_ is 150, then at FiO_2_ 50%, PaO_2_ is 75 mmHg, and at FiO_2_ 100%, PaO_2_ is 150 mmHg. The current results also demonstrated that the resupination FiO_2_ was higher in non-survivors and showed the highest inverse correlation with mortality.

The strengths of the current study include the use of granular data from a large multicenter database and a rigorous and comprehensive modeling approach to define the 28-day mortality following resupination based on the principles of oxygenation, dead space ventilation, pressure, and respiratory system compliance. The statistical methods used in this analysis were not based on clinical inference and, thus, do not imply a causal relationship between these variables and the outcome. However, the most consistent parameters were PaO_2_/FiO_2_, physiological dead space, and dynamic lung compliance at resupination, all of which are clinically relevant in routine practice. Finally, the Dutch Data Warehouse is one of few EHR databases that includes body position parameters with timestamps.

This study had several limitations. First, the present study was a retrospective analysis of a prospectively collected database, which might be problematic when used to build machine learning models to predict outcomes. Various biases inherent in observational data and inaccuracies and inconsistencies in medical records can distort a model’s understanding of the relationships between variables. Second, the finding that improvements in PaO_2_/FiO_2_ and other ventilatory parameters at resupination were prognostic factors may be due to the potential specificity of the COVID-19 phenotype^41,42^, making it difficult to generalize the results to ARDS due to other causes. However, the high ventilatory ratio, increased physiological dead space, and low lung compliance of the study patients were similar to those observed in patients with non-COVID-19 ARDS. Third, the sample size was relatively small for machine learning models, which might be more susceptible to overfitting, random variation, and outliers. The measurement frequencies varied for several variables, limiting data availability. Fourth, the MV or sedation protocols were not standardized, and the decision to initiate or discontinue prone positioning was left to the discretion of the attending physician, which might have introduced additional bias. Fifth, excluding proning patients without documented resupination may potentially introduce immortal time bias when measuring outcomes from the initiation of MV. Moreover, extended prone positioning (> 24 h) was not evaluated. Sixth, ARDS severity was not classified according to the PaO_2_/FiO_2_ thresholds specified in the Berlin criteria^20^.

In conclusion, survival after prone positioning in patients receiving MV can be stratified by physiological responses after resupination using novel machine learning techniques, even though the predictive performance is moderate. To increase clinical applicability and improve generalizability, future studies should collect and externally apply larger datasets. Furthermore, simple and feasible models to predict the response to prone positioning should be developed to allow the accurate identification of responders and timely adoption of other possible treatment strategies for patients who do not respond to pronation.

## Supplementary Information

Supplementary Information.

## Data Availability

All data generated or analyzed during this study are included in this published article (and its Supplementary Information files).
