# Supplementary material for: Respiratory physiology after resupination following prone ventilation to predict 28-day mortality in mechanically ventilated patients: a machine learning analysis
Source: Sci Rep. 2026 Feb 10;16:8188. doi: 10.1038/s41598-026-39336-3 (PMC12963402; doi:10.1038/s41598-026-39336-3)
Supplement: Supplementary file 1 — Supplementary Information. [file 41598_2026_39336_MOESM1_ESM.pdf]

Supplementary Information

Respiratory physiology after resupination following prone ventilation to predict 28-day mortality in mechanically ventilated patients: a machine learning analysis

Scientific Reports

Lada Lijović[1,2], Tariq A. Dam[1], Moon Seong Baek[3], Tae Wan Kim[3], Gyungah Kim[3], Paul W. G. Elbers[1], and Won-Young Kim[3] on behalf of The Dutch ICU Data Sharing Collaborators

[1]Department of Intensive Care Medicine, Center for Critical Care Computational Intelligence, Amsterdam Medical Data Science, Amsterdam Public Health, Amsterdam Cardiovascular Science, Amsterdam Institute for Infection and Immunity, Amsterdam UMC, University of Amsterdam, Vrije Universiteit, Amsterdam, The Netherlands. [2]Department of Anesthesiology, Intensive Care and Pain Management, University Hospital Center Sestre Milosrdnice, Zagreb, Croatia. [3]Division of Pulmonary and Critical Care Medicine, Department of Internal Medicine, Chung-Ang University Hospital, Chung-Ang University College of Medicine, Seoul, Republic of Korea.

Supplementary Table S1. Logistic regression analysis of factors associated with 28-day mortality.

Supplementary Table S2. Performance metrics for machine learning models for 28-day mortality prediction using 4-h post-resupination values after LASSO feature selection.

Supplementary Figure S1. LASSO regularization paths.

Supplementary Table S1. Logistic regression analysis of factors associated with 28-day mortality.

Variable	β coefficient
(Intercept)	–1.182
FiO_2	0.374
Physiological dead space	0.304
INR	0.187
Static lung compliance	0.155
Ionized calcium	–0.136
Arterial oxygen saturation	–0.149
Base excess	–0.180
Mean arterial blood pressure	–0.221
Lactate dehydrogenase	–0.328
Alanine aminotransferase	–0.397
Dynamic lung compliance	–0.441

FiO_2: fraction of inspired oxygen; INR: international normalized ratio.

Supplementary Table S2. Performance metrics for machine learning models for 28-day mortality prediction using 4-h post-resupination values after LASSO feature selection.

Model	AUC-ROC	Accuracy	Precision	Recall	F1-score
Logistic regression	0.711	0.771	0.619	0.317	0.419
XGBoost	0.719	0.618	0.380	0.732	0.500
Decision tree	0.677	0.732	0.486	0.415	0.447

AUC-ROC: area under the receiver operating.

Supplementary Figure S1. LASSO regularization paths. Each colored line represents the coefficient values of a particular feature (all 4-h resupination values) as the regularization strength (alpha) increases (or as $-\log_{10}$(alpha) decreases from right to left). The LASSO model was trained to predict 28-day mortality over logarithmically generated alpha values from 10^{-6} to 10, with five-fold cross-validation (cv = 5) and 10,000 iterations to determine the optimal alpha ($\alpha = 0.026$). The vertical dashed line indicates this optimal alpha value. At this point, features with non-zero coefficients (listed as “Selected Features”) are retained, and features with coefficients reduced to zero (listed as “Discarded Features”) are eliminated.

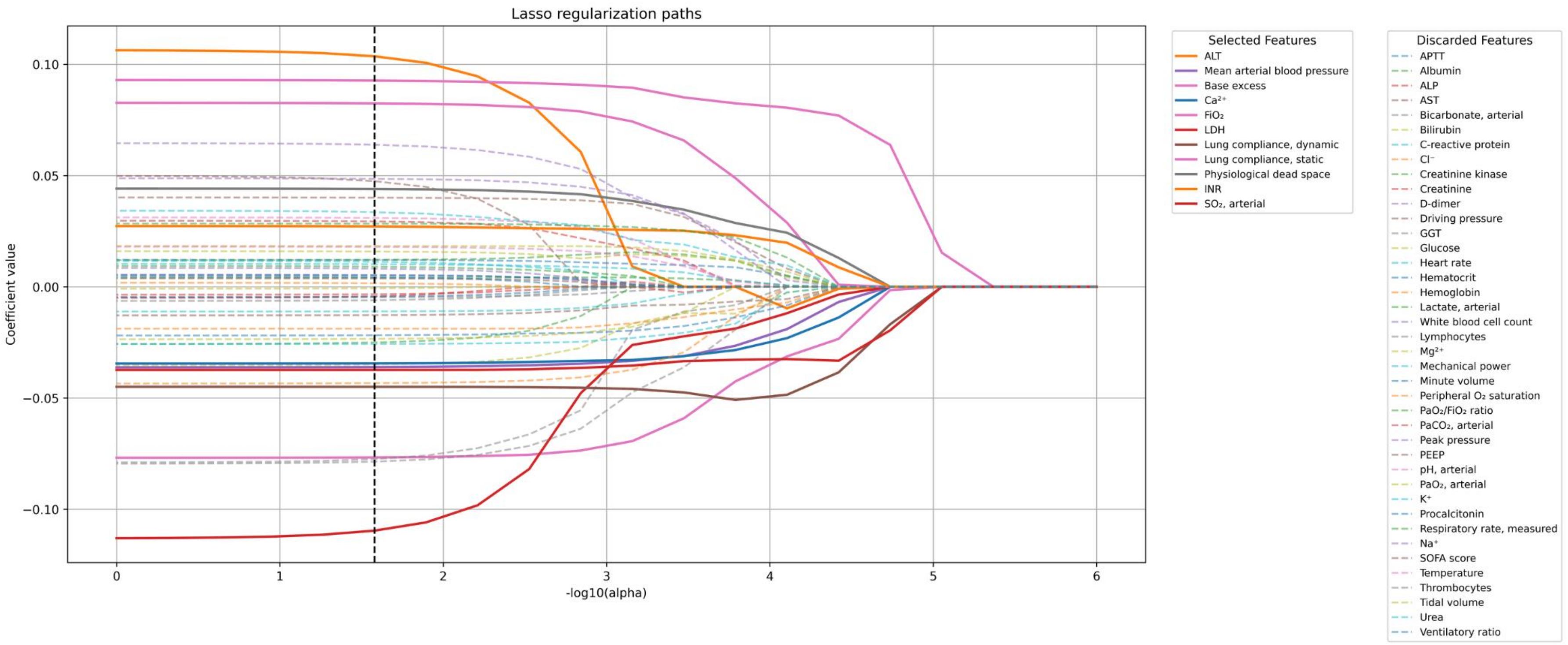

ALP: alkaline phosphatase; ALT: alanine aminotransferase; APTT: activated partial thromboplastin time; AST: aspartate aminotransferase; FiO_2: fraction of inspired oxygen; GGT: gamma-glutamyl transferase; INR: international normalized ratio; LDH: lactate dehydrogenase; $PaCO_2$: partial pressure of arterial carbon dioxide; PaO_2: partial pressure of arterial oxygen; PEEP: positive end-expiratory pressure; SO_2: oxygen saturation; SOFA: Sequential Organ Failure Assessment.
